# A Reference Model for Monitoring IoT WSN-Based Applications

**DOI:** 10.3390/s16111816

**Published:** 2016-10-30

**Authors:** Juan Vicente Capella, José Carlos Campelo, Alberto Bonastre, Rafael Ors

**Affiliations:** Instituto ITACA, Universitat Politècnica de València, Ciutat Politècnica de la Innovació, Camino de Vera s/n, 46022 Valencia, Spain; jcampelo@disca.upv.es (J.C.C.); bonastre@disca.upv.es (A.B.); rors@disca.upv.es (R.O.)

**Keywords:** Internet of Things, Wireless Sensor Networks, open monitoring architecture, monitoring platform

## Abstract

The Internet of Things (IoT) is, at this moment, one of the most promising technologies that has arisen for decades. Wireless Sensor Networks (WSNs) are one of the main pillars for many IoT applications, insofar as they require to obtain context-awareness information. The bibliography shows many difficulties in their real implementation that have prevented its massive deployment. Additionally, in IoT environments where data producers and data consumers are not directly related, compatibility and certification issues become fundamental. Both problems would profit from accurate knowledge of the internal behavior of WSNs that must be obtained by the utilization of appropriate tools. There are many ad-hoc proposals with no common structure or methodology, and intended to monitor a particular WSN. To overcome this problem, this paper proposes a structured three-layer reference model for WSN Monitoring Platforms (WSN-MP), which offers a standard environment for the design of new monitoring platforms to debug, verify and certify a WSN’s behavior and performance, and applicable to every WSN. This model also allows the comparative analysis of the current proposals for monitoring the operation of WSNs. Following this methodology, it is possible to achieve a standardization of WSN-MP, promoting new research areas in order to solve the problems of each layer.

## 1. Introduction

The Internet of Things (IoT) can be defined as an infrastructure of interconnected objects, people, systems and information resources together with intelligent services to allow them to process information of the physical and the virtual world and react accordingly [1]. IoT is also defined by the Organisation for Economic Co-operation and Development (OECD) as the convergence between Information and Communications Technology (ICT) and the economy on a grand scale [2]. As an enabling technology that provides support of existing and new applications, services and devices, the impact of IoT on day-to-day life of people and its environments may not be bounded at this time.

One of the main characteristics of IoT is that the objects must be context-aware. Many IoT applications require that the “things” know their environmental conditions, and adjust their behavior to them and to the other objects that are nearby. In this way, Wireless Sensor Networks (WSNs) can be considered the extension of the Internet toward the physical environment in the IoT, and thus they are one of the most valuable parts of any IoT system. WSNs are formed by many small devices, called motes, that are able to acquire information about their environment through sensing elements called transducers, process the obtained data and transmit it to a data center in order to store and collaboratively process it.

Thus, WSNs correct behavior is fundamental for the correct operation, and maybe the future success, of IoT applications. However, although WNS designs show good performance and behavior when being evaluated in controlled environments, experience has shown that their deployment in real environments results in poorer performance and behavior [3]. It is therefore necessary to improve this behavior, and for that, to have more accurate information of the internal WSN operation [3,4,5]. This information may be obtained by the utilization of appropriate tools, the so called WSN Monitoring Platform (WSN-MP) being the most suitable.

A WSN-MP is a distributed system which monitors the operation of a WSN, by means of a set of devices and/or modules which acquire information from different elements of the WSN (from the motes, wireless network, gateways, and even communication channel), and then collects, analyzes and presents it to the user to provide internal information about the WSN’s operation. Many efforts have been carried out to debug the operation of WSNs, but as explained in the next section, those efforts have resulted in specific tools that only can be applied to specific WSNs, while cooperation between heterogeneous WSNs is a key issue in IoT applications.

The use of WSN-MP may help in all the states of the life-cycle of a WSN. WSN researchers may use a WSN-MP to perform comparative analysis on new proposals. Designers may select the most suitable techniques for the requirements of any application. When deploying a WSN, the enhanced debugging capabilities added by the WSN-MP are unbeatable. The deployment is much easier when the correct functioning of the motes can be verified in-situ. During operation, malfunctions may be diagnosed without stopping the system, and the redesign of the system can use more detailed information about current functioning. In addition, these tools can become fundamental in the standardization and certification of applications based on the WSN.

With these goals in mind, WSN-MPs are an increasingly important research line, a fact corroborated by the publication of a large number of papers on this area in the scientific literature. The study of these proposals has shown the great disparity of different WSN-MPs and the lack of methodology for their analysis and design, which leads to the fact that each proposal uses different ad-hoc solutions. Additionally, we conclude that there is a set of different issues that every WSN-MP must solve to be functional, and the different solutions of these problems could be combined in order to obtain the appropriate characteristics for the desired Monitoring Platform. 

From the authors’ point of view, this situation is similar to those of computer networks, which began as a set of ad-hoc systems that did not follow any standard. The spectacular advances achieved in this field had been partially possible because of the adoption of a universally accepted architecture that promoted the use of standards. Following this approach, many researchers and standardization organisms have promoted the standardization of WSNs in the last years [6,7,8,9,10]. Now, we advocate for the standardization of WSN Monitoring Platforms (WSN-MPs).

Considering the example of computer networks, the aim of this paper is to propose a new reference model for WSN-MPs, to establish a systematic methodology for the analysis, design, implementation and operation of monitoring platforms on sensor networks. This model allows the comparative analysis of WSN-MPs, and offers a common environment for the design and standardization of new platforms [5]. It is based on decomposing the monitoring process into different layers, identifying the responsibilities and establishing the services, protocols and interfaces needed to accomplish them. This approach offers several new interesting features, such as flexibility (adaptable to any type of system or application), simplicity (due to layer division), universality (foreseeing any system), and adaptability (able to follow the future evolution of WSNs and their WNS-MPs). This model could serve as first approach to achieve a standardization of WSN-MPs.

If WSN-MPs’ design and implementation were to follow a standard, a rigorous and systematic approach to the characteristics of WSN would be possible, as far as it can be granted that the WSN-MP used when analyzing that both WSNs have the same properties, and thus certify their operation. This point becomes critical for IoT applications as data producers and consumers are decoupled. On the other hand, the implementation of new WSN-MPs may be simplified, building new monitoring platforms by combining off-the-shelf modules. Finally, a new standard reference model which has to be detailed also promotes new research areas to complete and develop the issues pointed in this article.

This paper is structured as follows: after this Introduction, Section 2 provides a review of the monitoring tools developed by the scientific community. Section 3 outlines the research design and methods used to achieve the main result of this work. This result, the proposed model, is presented in Section 4. As a validation phase of the presented model, Section 5 applies the model to the characterization of the WSN-MPs detailed in Section 2. Section 5 also uses the model to design a new WSN-MP. Finally, Section 6 summarizes the conclusions of this work.

## 2. Wireless Sensor Networks Monitoring Tools

Several tools have been proposed during recent years which facilitate the development of wireless sensor network applications. These tools assist engineers in different stages of implementation. In [11] an in-depth revision of the large number of different proposed tools is shown: simulators, emulators, data visualization tools, testbeds, debugging tools, reprogramming tools and network monitors. Frontiers between those groups are often vague and some of them can be catalogued in several groups.

More clearly, Schoofs [12] classifies wireless sensor network tools into two groups: pre-deployment tools and post-deployment tools. The first includes simulators, emulators, testbeds and debuggers, whereas the second includes traffic visualization tools, remote debuggers, programming tools and a set of active and passive strategies grouped together as monitors.

Table 1 additionally differentiates between the post-deployment tools that centralize the monitoring process and the ones that allow distributed monitoring. These tools can be classified [12], identifying the knowledge of the system (motes) about the monitoring process, as:

- Passive: No action is required by the mote and there is no interference with network operation. They rely on communications packets received at the network gateway or overheard within the network.
- Active: Motes are instrumented to make possible the monitoring process and the mote interacts with the monitoring tool. Network users interact with the motes to request or retrieve information about their state or about the network operation.
- Opportunistic: Motes are also instrumented to make the monitoring process possible but there is no interaction between the mote and the monitoring tools. The information about motes and network operation is sent by the motes to the network controller when it is necessary.

Thus, although not new, the concept of monitoring wireless sensor networks has adopted different names in designs and implementations over the years. Depending on the use and purpose of the deployment, different names have been adopted: monitor, sniffer, debugger, analyzer, diagnosis tools, etc. We advocate the use of the term monitor for such tools with a standardized and methodological classification. 

According to the literature, Ferrari [13] defines a monitor as a tool that quantifies the results of an observation. Svobodova [14] defines a monitor as a tool that facilitates analytic measurements necessary for performance analysis and evaluation. The name monitor reflects the fact that performance cannot be assessed from a snapshot measurement, but only after observing (monitoring) the system for the appropriated time lapse. The measurement process entails three main problems:

1. What information is pertinent to a specific measurement objective.
2. Where such information can be found.
3. How it can be extracted and processed.

Furthermore, a monitoring platform (as a distributed system) must solve some additional problems: data coherence between the different elements that form the distributed system, and the underlying communications subsystem to support collaborative processing.

To overcome the heterogeneity of developments in this research area and the lack of a complete classification and a systematic implementation methodology, we can outline the main characteristics of the most important developments.

Maybe the most widespread monitor is Nucleus [15]. Developed on TinyOS, it constitutes a complete network management system. Nucleus uses a service, which is independent of the data network, which allows the distribution of queries and collecting results. It also includes a system event logger, where variables and events to monitor are specified by the application programmer. Nucleus is the clearest example of active monitoring.

Sympathy [16] is the most cited example of passive monitoring. Sympathy analyses information flows received by the sink and it is able to infer faults and their probable origin using a decision tree. This is based on the premise that a sensor network application produces a predictable flow of traffic using periodic messages. Any changes in this flow indicate strange behaviors. The causes of these alterations can be deduced from the well-known characteristics of the traffic. PAD [17] assumes a similar approach, but each mote actively marks the packets sent with two bytes, which help to build an inference model to reconstruct the network topology in the central processing station.

The fault detector does not need to be centralized in the application gateway. The authors of Memento [18] propose a distributed system that sends periodic messages with information on their own state. These messages, bitmaps, are added along the way to the sink. In addition, each mote can detect faults in their neighbors if they fail to transmit a regular heartbeat. Thus, motes can transmit their view of network status, which can be corrected by their parents. Memento is an active monitor, but the intrusion is minimal.

Event-based networks, however, have no regular and predictable traffic. Bearing this in mind, the authors of Envirolog [19] proposed a system to record and replay events. The code of the mote is extended with annotations that mark which events should be stored in the non-volatile memory. Traces stored in this way may then be replayed upon demand, even at different speeds. Lightweight tracing [20] provides a path-encoding algorithm that produces highly compressed traces, which are stored in non-volatile memory. In NodeMD [21] a mote checks for faults, reporting them by sending a trace that stores the sequence of events that led to the failure, and automatically quarantines itself, entering an interactive mode to allow the network manager determine the causes of failure. PDA [22] proposes a mechanism called passive distributed assertions that allows developers to detect failures and provides hints on possible causes. PDA allow a programmer to formulate assertions over distributed node states using a simple declarative language, causing the sensor network to emit information that can be passively collected and evaluated to verify the assertions hold. Reference [23] presents FAMoS: a flexible active monitoring service to collect wireless sensor network traffic volume and distribution data. The service is provided with limited overhead and it is applicable in many contexts. Each node locally collects data about network traffic and then periodically transmits the data to a back-end for further analysis and processing.

All these cases involve some sort of intrusiveness in the system: the monitored application is not identical to the one that will be executed. This can lead to a ’probe’ effect: by removing the monitoring system (all resources are required in real installations) the system behaves differently and our initial premises may be invalid. Also, the instrumented code to provide information to the monitors can introduce new bugs.

Some proposals try to minimize the intrusion degree using the debugging port of sensor nodes: FlashBox [24] is a Flash-based system to record non-deterministic events in embedded systems that can aid in crash investigation and replay. It is a minimally invasive monitoring system. The Aveksha platform [25,26] provides tracing of events and breakpoints for a single node co-locating a debug board with the sensor node under test. Minerva [27] extends this approach by connecting the debug board to a test observer which is part of an out-of-band backbone network (Ethernet).

Spi-Snooper [28] integrates hardware and software in a hybrid approach. The hardware architecture brings the sensor node and the monitor together in a single unit in a transparent manner. The monitor spies on the SPI interface used to connect the sensor node to its radio module. The software architecture has two operation modes: active and passive. In passive mode the monitor—called the co-processor—mainly logs the communication through the SPI bus and checks some node data. In active mode it assumes the control of the SPI and the radio interface. However, techniques like this, based on external radio modules interconnected with the main microcontroller by means of some bus (like SPI) cannot be used in sensor nodes with built-in radio modules (as is usual nowadays) as data transmitted cannot be captured by external hardware and, additionally, the built-in radio module cannot be controlled from an external unit. 

In [29] an active low intrusion hybrid monitor for WSNs developed by the authors of this paper is presented. It consists of two elements: a software monitor running in observed ARM-based sensor nodes, and a monitor node attached to the latter which receives software traps issued by the former, in order to obtain relevant information about node operation. Several communication physical interfaces between monitor node and sensor node were evaluated according to intrusion, transmission time and power consumption. This preliminary implementation was a valuable experiment to validate and complete the reference model presented in this article.

On the other hand, “passive monitoring systems” means access to network traffic without interfering with the networks. Monitors are distributed near the networks using different radio or channel routes. SNIF [30,31] employs a sniffer over the communications channel, which snoops the data network, and another channel (Bluetooth) transmits this information to a central station. The central station analyses the received traffic and generates debug information from relationships between data. Unfortunately, this analysis relies too heavily on assumptions about the underlying data transmission protocols.

A similar approach is taken by PIMOTO [32]. In this case, the monitors are sniffers that transfer the captured messages to a central server through a PC gateway using a TCP connection. Traffic received in the server is analyzed using a general purpose sniffer program (Wireshark [33]), which is extended to interpret the wireless messages contained as TCP payloads. Other examples of passive monitoring are LiveNet [34], which analyses offline traces collected by sniffers or DiMo [35], where monitors, distributed throughout the network control the periodic transmission of heartbeats from motes. Halfway between active and passive monitoring DSN can be found [36]. This monitor uses a wireless deployment support network (DSN), where each mote is directly connected to another mote in the data network. In DSN the support network uses Bluetooth and their motes build a scatternet to reach a server. The main function of the DSN motes is to record events transmitted by the application motes through a serial channel, but can also serve as an infrastructure for remote programming, transmission of commands (RFP) and dynamic configuration of the monitoring. Reference [37] discusses Z-Monitor, a passive sniffer that captures network traffic (802.15.4 standard) and redirects it to a user-friendly graphical interface. A distributed sniffing mode allows that multiple standalone versions of Z-Monitor collect local pictures of the network and send the data through wireless or wired links to a Z-Server that maintains and shows the database (with an accurate synchronization among sniffers).

Unfortunately, traces collected using passive monitoring are limited in several aspects [38]: first the monitoring trace is incomplete because is hard to capture every transmission in the network due to packet drops caused by unreliable links, collisions, weak signals, limited resources, etc. Second, the information extracted from monitoring explicit traces, such as sender and receiver, is insufficient to evaluate network performance. For instance, the trace does not record whether each packet was successfully received by its destination. Trace merging [38] techniques and inference procedures are used to construct an enhanced trace of the network.

Can it be possible to analyze and compare all these tools from a standard and systematic point of view? Furthermore, can new designs, implementations and operation of monitoring platforms be guided towards production under standard classification and certification? All these questions could be answered positively if a reference model for these tools could be defined.

## 3. Research Design and Methods

As presented above, WSN-MPs’ developments are very diverse. Different ad-hoc approaches have been used to solve the many problems that arise when monitoring WSNs. A model is required to generate off-the-shelf components that may be combined to create new WSN-MPs. If applied, new WSN-MPs would be composed of layered modules with defined functions that communicate by means of standard interfaces, and thus reutilization in new WSN-MPs would be very easy. This model, if successful, should also permit a systematic approach to existing and future WSN-MPs to characterize their functioning and compare their features. This model is the result of the application of a scientific methodology, based on the well-known hypothetico-deductive model. It states that scientific inquiry must be proven by following the next steps:

1. Problem formulation, based upon previous experience: after an in-depth study of the different WSN-MPs, our experience in WSN monitoring has been applied to address the problems related to WSN-MP design.
2. Hypothesis formulation by means of a conjecture that may predict future results: from these observations, a proposal for WSN-MP layered model has been formulated. This model should consider all the necessities that a WSN-MP could deal with.
3. Deduce predictions from the hypothesis: if correct, the WSN-MP reference model must: (a) permit the creation of new WSN-MP by combination of off-the-shelf components. They may collaborate through standard interfaces to fit the requirements of any WSN monitoring campaign (for debugging, verification, certification or deployment purposes). The model must also: (b) allow a systematic study and characterization of existing (and future) WSN-MPs, in order to compare their features.
4. Verify that prediction accomplishes: the proposed model has been applied to both objectives, the results being shown in Section 5.1 and Section 5.2. From the application of the proposed model, the revision of hypothesis formulated in the second step was considered, and thus the model was changed and reevaluated.

As a result, a reference model that meets all the expected requirements has been proposed, and it is presented in Section 4.

## 4. Model Description

This section describes the proposed model starting with a model overview, followed with a deeper description of each one of its components and concludes remarking the main features that this model offers. As shown in the literature and mentioned above, every functional monitoring platform has to deal with some issues. Our proposal identifies these problems and classifies them into three categories:

- The first category makes reference to monitoring data. These data must be captured, analyzed and shown to the user in a meaningful way. The semantic meaning of the data is highly related to the application and the monitoring needs.
- Once obtained, data must be expressed in a convenient way to ensure the integration of data provided by heterogeneous sources. Some issues related to this deal with a common time base and capturing conditions.
- As the monitoring platform is distributed in space, all the data obtained must be centralized and stored to ensure a global comprehension of the system functioning.

**Figure 1:**
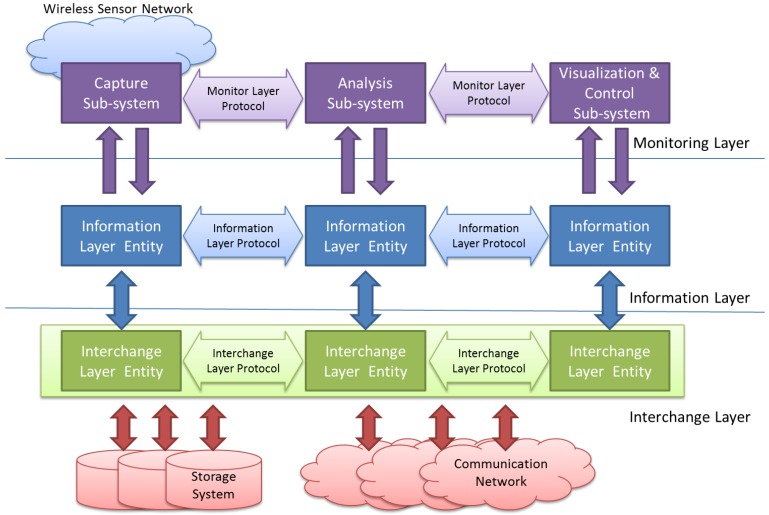
Proposed model.

Keeping these different—and usually independent—problems in mind, the proposed model is composed by three layers, as shown in Figure 1:

The Monitoring Layer will be located at the upper level of the model. This layer is in charge of all the issues related and specific to the WSN under observation. It must deal with the definition of what should be monitored, how this information must be acquired and the way it has to be processed and shown to the user.

The Information Layer is located under the Monitoring Layer. It must provide the support needed to code the obtained information in a standard way. This level also deals with timing issues, which includes when the information must be captured (triggering) and stores this time value into obtained data (time stamp).

Finally, the Interchange Layer allows the information captured in different points alongside the monitored WSN to be transferred and stored. Upper layers will retrieve this information to be analyzed and/or visualized by the Monitoring Layer.

The model must locate the interfaces between each pair of adjacent levels, obtaining in this way layer-independency, portability and interoperability between components from different sources (researchers, manufacturers, developers, etc.). It is also necessary the definition of a set of protocols to support the communication needs inside each layer. The standardization of these protocols will open the possibility of universal reuse of off-the-shelf components. Each of these layers is detailed in the following sections.

### 4.1. Monitoring Layer

#### 4.1.1. Layer Overview

This layer deals with the issues related to capture, analysis and interpretation of the information related to the monitored system functioning. As shown in Figure 1, it is composed by three subsystems. *Capture subsystem* acquires data from the observed system. These data are processed by the *Analysis subsystem*. This *Analysis subsystem* is in charge of joining and sorting the information of all captures in the monitoring platform, and processing it, applying the appropriate information algorithms (indeed using Artificial Intelligence techniques) to extract the required conclusions. 

Finally, these results must arrive to the *Visualization & Control subsystem*. The interface of the user is provided by this module, showing the required values in a useful way. This subsystem may also receive indications, instructions or parameters from the user, with the purpose of modifying the functioning of the Monitoring Platform, in both capture and analyze subsystems. It should be done in a friendly manner and offer a powerful yet simple interface.

#### 4.1.2. Components

At this level, the main source of data is the “Monitoring Data Point” (MDP). It is not a physical point but rather a logical one, corresponding to the information to be obtained. For instance, a MDP may be a communication routine which generates a software trap in a “*successful_transmission*” event. Other example could be a MDP to measure the remaining battery in a mote.

In order to obtain the MDPs’ data, it is necessary to use so-called “probes”, which acquire these data from the observed system. For instance, a probe may be a trap collector module (software probe) waiting for a “*successful_transmission*” event. Or it may be the circuit (hardware probe) that measures the remaining battery life in a mote.

**Figure 2:**
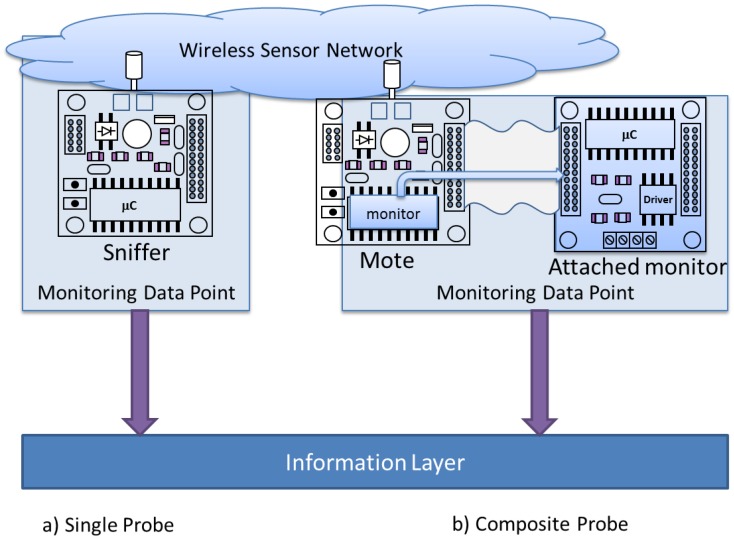
Example of (**a**) Single probe and (**b**) Composite probe.

It is necessary to define at least one probe for each MDP. Probes (Figure 2a) may consist in a single capture point, as the located in a sniffer [32,34] or a test bed which physically measures the signals on a PCB. There also exist composite probes [36] (Figure 2b), being composed by two or more elements. In this case, at least two elements must be present to acquire the desired data: an element in the monitored mote and other correspondent element located in the monitor mote.

The MDP is the main concept of the Monitoring Platform. Data acquired by probes usually consists of a trace of events, which reflects the behavior of a single MDP in the WSN. In this line, the WSN-MP main function is to collect all the relevant MDPs’ traces in an efficient way, joining and synchronizing them to study the behavior of the WSN.

This methodology abstracts the monitoring requirements from implementation issues, hiding part of the technical complexity of a monitoring platform design. This way, the designer can focus mainly on what it is intended to be captured and how to process and display that information, following the desired specifications.

The interchange of information inside this layer must be performed by means of the Monitoring Layer Protocol (MLP), which must ensure the coordinated subsystems operation. This protocol is a crucial point of the standardization and reutilization of elements of the monitoring tool.

Most of existing proposals do not implement analysis or visualization subsystems. This way, there is not a protocol to communicate data from capture subsystem. In other cases, the storage format is the only interchange route. Other tools, such as [34,39,40], make a huge effort to process obtained data, using their own protocol. Unfortunately, all these works were made without following any standards, and then is not possible to reuse them directly.

The WSN-MP designer is responsible from the definition of each MDP to study, as well as the different probes which must be associated to them. The combination of an MDP and its associated probes must define which data monitors (the MDP), and how these data is monitored (its associated probes), by defining hardware and software tools, elements and procedures.

Acquisition system inside the probes will produce a stream of raw, unformatted data. The lower layer (Information Layer) is responsible from applying the appropriate actions to express this information in a common standard way.

### 4.2. Information Layer

**Figure 3:**
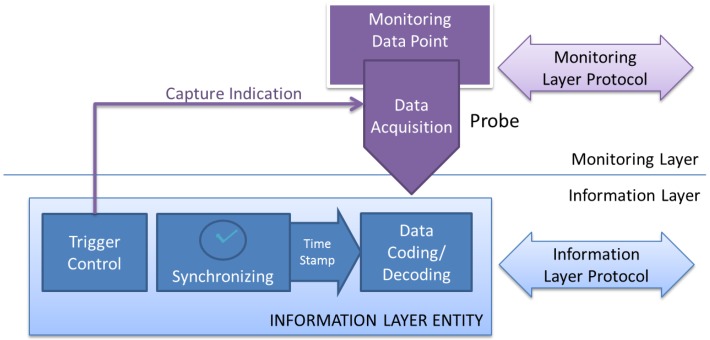
MDP, Probe and Information Layer Entity.

This layer is responsible for several functions. It is in charge to homogenize the probes’ captured data, delivered by upper layer in raw mode (without formatting). It is also concerned with the timing of the capture, applying a time stamp mechanism which makes possible the temporal sorting of events proceeding from different sources, in the same mote or in another mote of the monitored WSN. Finally, it is also responsible for the capture’s trigger, providing to the Monitoring Layer the necessary trigger indication, as shown in Figure 3.

This level offers its services to the upper layer, in such a way that it interchanges information with it (accepts information from capture entity, and delivers it to the analysis and visualization entities). Similarly, it uses the services offered by the lower layer (interchange layer) to move and/or store the information between Information Layer Entities. Virtually, this communication must accomplish the so called *Information Layer Protocol*, which must provide the support for the services of this layer. These services are discussed with more detail below.

#### 4.2.1. Data Format

To achieve greater universality, the acquisition mechanism of each probe delivers its data in raw format, in continuous streams of bytes. To convert these raw bytes into significant information, each probe must define its own data coding/decoding element. These elements must apply a syntax description, common to all the probes in the monitoring platform, to make its data compatible with the rest of the system. The final aim of this layer is the standardization of the description language in order to universally unify the WSN-MP specification, which would lead to the previously mentioned advantages.

Each significant data unit obtained must be expressed in a standard way. Each piece of information obtained should include what information reflects, where has been obtained, when has been captured (time stamp) and why is relevant (trigger conditions).

For instance, description languages such as XML may be highly adequate for this purpose. Each piece of data may be contained in a meaningful XML record. The Information Layer Protocol must allow the Standard data interchange. As an example, the events captured in PIMOTO [32] may be described in a XML record as follows:

  <trace date=”date of the trace” monitoring_experiment=”monitoring experiment identification”…>
     <event monitor_address=”MAC address of monitor node” source_address=”BMAC source address”
      destination_address=”BMAC destination address” 
      data_lenght=”Lenght of captured data” type=”application type” 
      time=”global time in which event occurs” ……. >
     data_collected 
    </event>
    <event … >data_collected </event>
      ·······
    <event … >data_collected </event>
  </trace>


Additional information about each event (as trigger mode, location, and so on) shall be added to configure a standard and universal information protocol.

#### 4.2.2. Synchronism

The main improvement obtained from the use of a WSN-MP over traditional monitoring tools is the possibility of correlate the events that happen in different MDPs, sort them into a unique time line and extract the results defined by the monitoring user.

From the study of the existing platforms several conclusions may be obtained. Most of the evaluated ones have not considered this issue [16,35], while other platforms [32,34,36] implement elemental synchronism mechanisms which provide low precision results. From these, some of them [32] charge the weight of synchronism in the analysis subsystem, simplifying the time stamp synchronization algorithm. Nevertheless, others [36] can simplify the analysis using a more complex time stamp algorithm. 

A synchronization algorithm, such as [41] or [42], should be considered at the Information Layer Protocol, to keep the same timeline in all the MDP data from every monitoring mote. Usually, the accuracy of the analysis will depend on the precision offered by the synchronization algorithms. These allow, not only the correct timeline characterization, but also may be fundamental in order to analyze the time constrictions of the system, which is a key characteristic of time critical applications. In this way, last advances in this area [41,42,43] can be applied at this layer to obtain the best results. The used synchronization algorithm must be part of the Information Layer Protocol, which must offer the user the possibility to select the most appropriated synchronization protocol for the characteristics of the monitoring application. 

#### 4.2.3. Triggering

Usually, information collected by a monitoring system is captured when some relevant event occurs in the monitored system. For instance, a software trap informs the monitoring system that a packet communication has been done successfully, or periodically informs of the remaining battery in the mote. In other cases, to characterize the global system functioning it is necessary to perform a synchronized capture in several motes when some events happen in other motes or even following external conditions defined by the user. 

However, sometimes the event that causes the acquisition of information is not generated by the monitored system. For instance, it may be of interest to measure the battery level when a special condition happens, i.e., when the mote is transmitting, or even triggered by a combination of several factors, some of them being system-dependent and other programmed by the monitoring user. The generation of a trigger signal, which indicates when the information has to be captured, may follow several policies:

- Periodical: the trigger signal is generated with a predefined period. The Information Layer may synchronize the trigger signals in different probes in the system, even when those probes are located in different motes, in order to get a simultaneous snapshot of the system.
- On user query: the visualization element may receive from the user the order to capture the data from any probe in the system, and provide this information to user interface.
- In function of other variables: the information can be captured when one or several rules become true. These rules may involve events, thresholds of measured variables, time conditions, etc., all of them in the same or in other mote.

The Information Layer Protocol must allow triggered operation. This way, the trigger condition of each probe must be selectable. Also, the states which may cause a trigger event in other monitor nodes must be disseminated during runtime. The trigger operation may define a set of rules to define trigger conditions, and variable and event notification to allow coordinate triggering. Those rules may be active at the same time, being combined for a flexible and powerful system analysis.

The Information Layer Protocol also must grant the deliverance of all the data to the Analysis and Visualization & Control sub-systems. This is achieved by means of the Interchange layer services. 

### 4.3. Interchange Layer

A monitoring platform is also a distributed system itself. All data managed by the information layer are spatially dispersed, and usually have been captured at different moments. They may be stored in non-volatile memory to be downloaded (or even physically transported) later. It is necessary to guarantee that the information communication between system components is correct. Issues related to data storage and communications (if exist) must be considered at this level. The Interchange layer must provide the information layer from those services required to fulfill its functions. 

This layer can offer the distributed data storage service to the upper layer, making use of one or more communication networks. Monitored data is stored in this layer in a structured way, being able to deliver the stored data when it is required by the Analyzer subsystem or by the Visualizer subsystem. 

**Figure 4:**
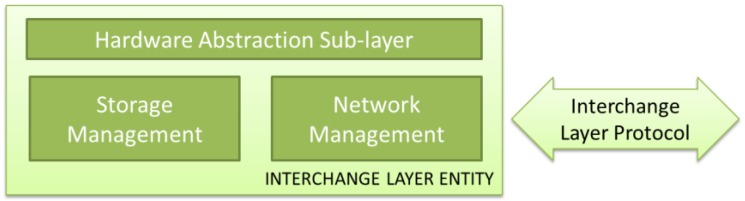
Detail of Interchange Layer Entity.

Fortunately, communication and storage technologies have been developing its own standards in the last decades. The utilization of these standards, and future ones, relies in the definition of a sub-layer which can use the services offered by these implementations in an efficient way. In this sense, a Hardware Abstraction Sub-layer, which permits the adaptation to any of those existing standards to be applied, is required. Despite of not suggested, a WSN-MP designer may choose to implement its own non-standard network or storage technology, and integrate it with standard upper layer elements if the appropriate abstraction sub-layer is defined. An Interchange Layer Entity must provide, at shown at Figure 4, two groups of services:

- Network Management: this element has to deal with communication issues, such as device addressing, device discovering and routing between the different used networks.
- Storage Management: this element has to deal with distributed storage issues such as data coherence, data storing and data querying.

Like any other element of this architecture, the definition of standard interfaces makes possible the migration from different implementations, as far as detailed services and functions are provided. In this way, many systems may be applicable, from powerful communication networks (wired or wireless networks, public or private) to the simplest, such as data storage in non-volatile memories carried manually to a data center.

### 4.4. Proposed Model Features

Some of the main features obtained with this new paradigm are:

- Universality: there are a lot of proposals, and even several of them already installed, that may benefit from the monitoring advantages. In this line, the first premise of the monitoring network should be its universality. This means that the proposed architecture is able to be applied to any currently designed WSN-MP as well as in future designs.
- Adaptability: as far as the WSN-MP may be required to monitor a wide spectrum of different characteristics, the architecture is adaptable to the needs of each application. The model is capable of fulfill the requirements of different user profiles: designers, supervisors, standard verification offices, and even final users. The model is also applicable to different application scenarios: real-time applications, performance studies, reliability certification.
- Flexibility: the architecture supports the fact that, once installed on a system, it may be necessary to modify the characteristics of the WSN-MP to monitor other features or increase the detail of the previously obtained data. This may be necessary in monitoring campaigns, which starts by analyzing the system as a whole, and later focusing the research on the more relevant and specific aspects.
- Simplicity: due to the problem division in independent layers, this architecture simplifies both the comparative analysis of the existing platforms and the design of new WSN-MPs. The increased composability obtained by the use of standard off-the-shelf components (capture, analysis, visualization, triggering, synchronism, etc.)

New platforms implemented following this model will maintain the advantages of standardization (reuse and interchangeability of hardware and software, independence between layers, isolated resolution of each task associated with a layer, data intercommunication between platforms, etc.) without losing the necessary specialization, keeping the ability to be optimized for each WSN and the appropriated parameters to be monitored depending on the application characteristics. These desired characteristics may include parameters such as dependability, bounded response time, early detection of coverage loss and mobility management.

## 5. Analysis and Design Examples

To demonstrate the applicability of the proposed model, an example where it is used to cross-platform analysis and another where a design is outlined following the methodology proposed are presented.

### 5.1. Analysis

The proposed model can be used to analyze monitoring platforms in a structured and systematic way, and compare the characteristics of each tested platform, their strengths and weakness, identifying its improvement issues. The application of the model starts with the identification of what data should be acquired, by means of the definition of the relevant MDPs and their associated probes. After that, it is necessary to identify the analysis subsystem required, and the visualization subsystem, if considered. Once monitoring layer elements have been determined, it is necessary to study how information is coded after being captured, and eventually after being analyzed, to be presented to the user. Synchronization and Triggering mechanisms may be also present, and must be reflected. Finally, the different communication and store mechanisms must be identified. Following these steps, we have proceeded to analyze the platforms described from the scientific literature in Section 2. The results of the analysis are shown in Table 2.

This table shows that all the studied WSN-MPs deal with the previously mentioned problems in a different, non-standard way. They have not considered any reutilization of developed elements, so each WSN-MP started its module design from scratch. This table summarizes the features of the studied WSN-MPs, overcoming its differences and offering a comprehensive comparison of characteristics, despite of its hardware implementation and the characteristics of the observed WSN.

This qualitative analysis reduces all WSN-MPs to a common denominator. The problem to solve (the WSN to monitor and the monitoring campaign requirements) will define the applicability of each WSN-MP. Their weakness and strengths have to be evaluated in relation with this monitoring campaign. 

For instance, Table 2 would be useful to notice that all PIMOTO, LIVENET, SNIF and Z-MONITOR use sniffers as MDPs, thus they are not able to observe internal motes’ behavior. MEMENTO allows the observation of the internal state of the motes, but it requires a serial channel for transmitting its observations. Z-MONITOR may also use a serial channel, but offers the possibility of wireless transmission of results. If both node internal state monitoring and wireless communication are required, a compromise must be arisen.

On the other hand, Z-MONITOR offers a graphical user interface for visualization, whereas PIMOTO uses Wireshark as trace visualization tool and LIVENET has no visualization functions at all. This analysis does not qualify the WSN-MPs, but it may be used to select which monitoring platform would be more suitable for a specific WSN monitoring campaign. In our case, it may be considered the advantages of using an ad-hoc graphical user interface (offered by Z-MONITOR) versus a common visualization tool such as Wireshark (offered by PIMOTO), less powerful but more flexible.

It also may be observed that some WSN-MPs have not implemented the features of all the layers. The designers of a WSN-MP may improve its proposals to provide services that other WSN-MP have already considered.

Finally, it would be possible to establish a ranking or score for each tool depending on how complete they are, that it is to say, how many features provide. A “completeness index” could be defined, that increases as the proposal offers more services, but this is not straightforward. In author’s opinion, a brief description of the features could be more correct, like “connection oriented” and “connectionless” attributes of TCP and UDP protocols in Transport Layer. Layer services cataloging could be a new research topic.

### 5.2. Design

Let us suppose that all the previously studied WSN-MPs followed the proposed layered architecture, after the full specification of the interfaces between layers. A wide collection of off-the-shelf components is available in public libraries, ready to be included in a new WSN-MP. As an example of how this model can systematize (and help to) the design of a monitoring platform the following scenario is considered:

A research group is working in the development of a new WSN routing protocol with the main objective of increasing the system reliability and keep compatibility with an IoT platform. To compare its performance with the existing protocols and obtain information on which aspects need to be improved, this group considers the utilization of a WSN-MP to monitor and compare the behavior of the different protocols to be evaluated. Obviously, the development of this WSN-MP must be as simple, low cost and optimized as possible (the challenge of the group is the research in reliable WSN protocols and not the development of WSN-MPs). Then the first option will be the use of a commercial one but: (1) it does not exist and (2) it must be particularized for this purpose. Then the best option shall be a “modular” WSN-MP composed by combining off-the-shelf pieces of software from other WSN-MP, following the model proposed in this paper. Each layer must be defined in a systematic way, and then off-the-shelf components that fulfil the specification may be combined to provide the desired features. The process is described below.

#### 5.2.1. Monitoring Layer

The first step to design the WSN-MP applying the proposed model consists in determining the relevant data for the desired study and how they can be collected. In our terminology, this means the identification of the required MDPs and the definition of the accurate probes to measure them. 

The relevant information for our example consists of the set of events generated in each of the motes of the WSN when a message is sent. Each time a mote is able to send a successful message and its ACK is received, a *Transmission_success* event is generated. On the other hand, a message can fail. A message is considered to fail in two situations; each one causes an associate event to monitor:

- Communication error: The message transmission must be followed by an Acknowledge message (ACK) sent by the destination mote to the source mote. If not received, the message is sent again several times. If no response is obtained from the mote, the message is discarded, and a *Transmission_error* event is generated.
- Route error: Each transmitted message has a hop-counter, which is decreased every time the packet is retransmitted. When the counter reaches zero, the packet is discarded and a *Routing_error* event is generated.

The first MDP considered will be the *Transmission_MDP*, which can generate one of three possible events: *Transmission_success*, *Transmission_error* and *Routing_error*, as described. 

The Monitoring Platform may also consider the use of *sniffers* to improve the knowledge of the WSN`s behavior. This *sniffer* will be the second MDP, called *Channel_MDP*, and will generate a *Captured_frame* event when a packet appears in the wireless communication channel.

The state of the mote is also considered relevant. Malfunctioning motes may cause errors, which are unrelated to the routing protocol but impair its performance. This way, the last MDP considered consists in the state of the mote, reflected by *State_MDP*. The internal state of the mote is evaluated from obtained values from internal registers from the microprocessor by a software routine. This routine must send this state periodically, generating a *Mote_state* event. Applying the proposed model, the *Capture subsystem* defines the probes to consider for each of the previously defined MDP’s. The first MDP (*Transmission_MDP*) can be measured by means of a hybrid composite probe implemented by means of two elements:

1. A piece of software, which is in charge of receiving the software trap included into communication routines, calculates the hash of the payload data and sends it through a predefined hardware interface into…
2. An attached monitoring mote, located next to the sensor mote, which receives the trap and its associated info.

The *Channel_MDP* is measured directly by using a probe implemented by means of the *sniffer* mote.

Finally, *State_MDP* is captured by the monitoring mote. If no *Mote_state* event is received through the predefined hardware interface for a defined time-out, *Mote_state* is assumed to be *out_of_service*.

All data collected by the probes will arrive to the *Analysis subsystem*. In this subsystem the different events must be chronologically sorted, in order to obtain a time-sequenced trace, and several algorithms must be executed over this trace to summarize the WSN’s behavior:

1. *Transmission_error*, *Transmission_sucess* and *Routing_error* events must be evaluated to determine the reliability of each link between motes, of each mote in the network and the overall network reliability, related to the routing algorithm used.
2. By inspecting the source and destination addresses of each packet, it is also possible to follow the evolution of the packets in the network, and study how the routing algorithm has evolved to avoid links and/or motes with low reliability.

This information must be sent to the *Visualization subsystem*, which will show the obtained results in a friendly way, usually by means of a graphical web-based user interface.

The proposed Monitoring Layer could be obtaining by combining the Capture Subsystems of EnviroLog (software traps) and PIMOTO (sniffers), the Analysis Subsystem of LIVENET combined with the algorithms on SNIF and a Visualization Subsystem similar to the used in PIMOTO combined with MEMENTO’s Failure Detectors. The availability of such off-the-shelf components would permit the fast integration of this features in a single Monitoring Layer implementation.

#### 5.2.2. Information Layer

In this layer, following the proposed model, three main issues have to be solved:

- The data format interchanged between the different elements must be based on standard data formats, from the byte coding to the semantic meaning of each data. In this way, the best option seems to be a XML-based data codification format. In this representation, each event provided by Monitoring Layer will be reflected by an XML record as shown in previous example (Section 4.2.1).
- It is necessary the establishment of a common base time. In this case study the synchronicity may be obtained by implementing the proposal of [42] which uses max consensus to compensate for clock drift and average consensus to compensate for clock offset. With this approach is possible to achieve a global synchronization just using local information. This solution has the advantage of being totally distributed, asynchronous, and robust to packet drop and sensor mote failure.
- Finally, a *trigger* mechanism is required to acquire the information at the convenient moment. Some of the events (i.e., those related with transmission) are independents, and are captured at the moment they occur. On the other hand, when a *Comm_error* or a *Route_error* event is detected, the *mote_state* of the destination mote of the lost packet is very relevant. In this way, the appropriate cross-trigger mechanism has to be implemented.

Of course, Information Layer Protocol must be ready to support all three functions: transmission of the XML records, synchronism information and trigger conditions definition and event notification. It is remarkable that these functions would be achieved in a very easy way if Information Layer implementation of SNIF (Synchronism and Time Stamp) could be combined with the PIMOTO metadata and PAD’s implementation of Packet Parsing. The availability of Information Layer off-the-shelf components would permit the communication between such modules, providing a full Information Layer implementation.

From the authors’ point of view, this layer is the key for the standardization of WSN-MP’s. The establishment of a complete Information Layer permits the definition of the whole protocols and services in a standard way.

#### 5.2.3. Interchange Layer

As previously described in the model, the main function of Interchange Layer consists in moving the information from capture elements to analysis element, and then from analysis element to visualization element. As previously described, it is necessary the definition of a Hardware Abstraction sub-layer which permits the use a standard communication and storage systems. An implementation of the Hardware Abstraction Sub-Layer may offer, as mentioned, the discovering of existing monitoring devices (attached motes and sniffers), which inform the system about their capabilities and transparent data storage.

In our example, communications could be implemented by means of wired networks. The sniffers are static and are powered with a wired network, so an Ethernet wired network may be used to connect these motes with the analysis system. Monitor motes attached to the motes of the WSN may also be provided with an Ethernet adapter. Communication between Analysis and visualization subsystem may also be implemented by the Ethernet network. 

The transparent storage could be implemented by means of a SQL database located in the same Analysis Subsystem. Motes may introduce its information by means of SQL commands over a TCP/IP connection. The implementation of this Interchange layer would be easy by combining PAD and LIVENET Interchange layers. Off-the-shelf components that implement these features would communicate through standard interfaces to provide the required Information Layer implementation. The characteristics of this WSN-MP are summarized in Table 3.

This work will open a discussion about the appropriateness of each of the proposed layers, and reveals the need for the entire model standardization to guarantee its correct operation. However, it is clear the advantages of a reference model as proposed in this paper. It can also be considered a starting point for establishing a universally accepted standard.

Standardization organisms should be aware of the need of this environment where WSN-MP can evolve. For instance, ISO is doing a huge standardizing effort in WSN standards through its ISO/IEC JTC 1/WG 7 Working Group on Sensor Networks committee [9]. Maybe this committee should also take this task into account.

## 6. Conclusions

The Internet of Things has changed the way designers must deal with Wireless Sensor Networks. From ad-hoc, specific WSN deployments, IoT environments require that new WSNs will be able to integrate into a complete IoT ecosystem in a compatible way. The availability of appropriate monitoring platforms (WSN-MPs) is required to validate and certificate new WSNs’ behavior.

The bibliographical study of the different WSN-MP proposals has shown the need to dispose of a systematic methodology for the analysis and design of these systems. To cover this lack, this paper proposes a reference model that systematizes the comparative analysis of present and future WSN-MPs and aids in the design of new ones. The proposed model is based on the division of the monitoring issue into three layers: a Monitoring Layer that focuses on relevant information: data capture, analysis and visualization, an Information Layer the deals with information coherence: data format, synchronization and triggering and finally, an Interchange Layer that supports information management: communication and storage.

Examples of both analysis and design have been presented to demonstrate the applicability of the proposed model. In the analysis example, several platforms from the literature have been selected and analyzed. As a result, it has been corroborated that this methodology is easily applicable to any WSN-MP, in a flexible and standard way, obtaining all necessary information that allows easily extract its comparative characteristics. On the other hand, in the design example, it has been shown how, starting from initial specifications and applying the proposed model, it could be possible by combining off-the-shelf modules in an easy way, to obtain the design of a WSN-MP that meets those requirements.

Moreover, the WSN-MPs designed with this model cover all stages of the life cycle of a WSN. Additionally, this model can be used for future certification processes of WSNs in IoT-based applications. The proposed model is ready to be applied in the analysis, but in order to design new WSN-MPs it is necessary to continue with the specification of the interfaces between layers and the research and development of protocols and services of each layer. This will allow the development of universally accepted standards. New platforms implemented following this model will maintain the advantages of standardization (reuse and interchangeability of hardware and software, independence between layers, isolated resolution of each task associated with a layer, data intercommunication between platforms, etc.) without losing the necessary specialization, keeping the ability to optimize for the WSN and the appropriated parameters to be monitored depending on the application characteristics. These desired characteristics may include, beyond compatibility issues, parameters such as dependability, bounded response time, early detection of coverage loss and mobility management. 

The proposed model opens several research areas. In terms of standardization, interfaces and protocols must be defined. In terms of development, the best suitable options for each of the services must be selected. Finally, the commercial design and implementation of standard modules according to this model must be addressed.

## Figures and Tables

**Table 1:** Schoofs’ [12] tools classification lists.

Pre-Deployment Tools	Post-Deployment Tools	
	Centralized Monitoring	Distributed Monitoring
Software debuggers Software simulators Software emulators LowPAN Testbeds	Active and passive packet analyzers Logging & Tracing IP network utilities	Network self-diagnosis Remote-debuggers Packet sniffers & Visualization Distributed packet sniffers

**Table 2:** Systematic analysis of studied WSN-MPs.

	MONITORING LAYER				INFORMATION LAYER	INTERCHANGE LAYER
	*Capture Subsystem*		*Analysis Subsystem*	*Visualization & Control Subsystem*		
TOOL	MDP	Probes				
PIMOTO [32]	Air (Traffic)	Sniffer nodes	No analysis	Visualization in Real Time	Timestamp,	Bluetooth➔PC
					Metadata	
		2nd WSN-Bluetooth		WireShark	Radio packet	PC by TCP/IP➔Server
LIVENET [34]	Air (Traffic)	Sniffer nodes (*)	Merging data process: sniffer traces➔1 trace	No visualization, delivers trace files.	Timestamp	Ethernet
		Host (PC) attached to * (serial channel): raw format	Trace Analysis on the merged trace, based on several analysis tools			
DSN [36]	Target Nodes information	Data and event logging strings.	XML-RPC to access server database	Developer responsibility	Time Synchronization protocol for timestamp (DSN nodes)	Wireless Monitoring Network
		DSN nodes attached target nodes (wired)				
NODEMD [21]	Software faults	Algorithms to detect software faults	Fault diagnosis by the user	No visualization	Compressed event trace	Internal Memory of each node. Later, application wireless network.
	Event logging	Event logging system				
PAD [17]	Network communications dependencies	Packet Marking	Probabilistic inference model	Dependencies graph	Packet parsing (sink)	Application wireless network.
			Inference engine			
ENVIROLOG [19]	All-levels events	Software annotations		PC Java Tool: receive messages and display	PC Java Tool: encoding, injecting messages	Internal Memory of each node or SD.
DIMO [35]	Network topology,	Observer nodes	Message to the sink if no node heartbeat			Application wireless network.
	Node health status	Nodes must send heartbeat	Topology adaptation			
SYMPATHY [16]	Pre and post deployment all levels failures	Sympathy code added to the application. Snooping the channel	Sympathy Linux sink: failure detection and debugging	Sympathy Linux sink: log file		Application wireless network.
SNIF [30,31]	Network traffic	Sniffer nodes with two network interfaces. Passively observe	Flexible mechanism to decode overheard packets	Operators-SQL	SNIF nodes are time-synchronized: time stamp packets	Deployment support network (DSN): Bluetooth
			DSN Sink: Online analysis			
			Operators		Data stream: typed and time stamped	
MEMENTO [18]	Nodes status	Heartbeats Local status bitmaps	Failure detectors	Global aggregate results to a Gateway node: human readable	Aggregate result of nodes status bitmaps	2nd wired serial channel for collecting results
LIGHTW.T.T [20]	Control flow tracing	Software calls	Non volatile memory querying interface	Non volatile memory querying interface	Trace compression techniques	Non volatile Flash memory
PDA [22]	Node state	Code assertions	Back-end trace & merging messages into an ordered trace	Graphical user interface	Timestamp	Application wireless network/logging in the node/logging in sniffer nodes/2nd wireless channel
Z-MONITOR [37]	Air (traffic)	Sniffer nodes	Parsing functionalities of the bit-streams	Graphical user interface	Synchronization method based on NTP protocol	Local storage/2nd wireless or wired channel
FAMOS [23]	Network stack	Hooks	Back-end query subsystem	Database access		UDP➔ Back-end
FLASHBOX [24]	Non-deterministic events	ISR routines	No analysis	Trace flash file		Local Flash memory
AVEKSHA [25,26]	Trace events	On-chip debug event detection	No analysis	Event trace		USB➔ PC
MINERVA [27]	Register/memory access. Breakpoints and watchpoints	On-chip debugging mechanisms	No analysis	Phyton interface	Synchronization method based on NTP protocol	UDP➔ PC
SPI-SNOOPER [28]	Information transmitted and received by the node	Spies the SPI interface between microcontroller and radio module	Trace reconstruction	Trace flash storage logs	Timestamp	Flash storage & Application wireless network

**Table 3:** Sample WSN-MP characterization.

MONITORING LAYER				INFORMATION LAYER	INTERCHANGE LAYER
Capture Subsystem		Analysis Subsystem	*Visualization & Control Subsystem*		
MDP	Probes				
*Communication events:*	Composite probe:	Merging traces	Web-based visualization	XML records with TimeStamp, Metadata and Radio packet	Ethernet based platform
*Transmission_Error*					
*Routing_Error*	Attached monitor node (Traps)				
*Transmission_success*		Trace Analysis on the merged trace, based on several analysis tools			SQL database
*Captured_Frame*	Single probe: Sniffers				
*Mote_state*					
